# High-coverage targeted lipidomics could reveal lipid alterations and evaluate therapeutic efficacy of membranous nephropathy

**DOI:** 10.1186/s12986-022-00701-4

**Published:** 2022-10-12

**Authors:** Zhenzhen Lu, Conghui Liu, Qingqing Wu, Yueyi Deng

**Affiliations:** 1 Department of Nephrology, Longhua Hospital, Shanghai University of Traditional Chinese Medicine, 725 Wanping Road, Shanghai, 200032 China; 2 Shanghai Institute of Biochemistry and Cell Biology, Center for Excellence in Molecular Cell Science, Chinese Academy of Sciences, 320 Yueyang Road, Shanghai, 200031 China; 3 Key Laboratory of Systems Health Science of Zhejiang Province, School of Life Science, Hangzhou Institute for Advanced Study, University of Chinese Academy of Sciences, Hangzhou, 310024 China

**Keywords:** Membranous nephropathy, High-coverage targeted, Lipidomics, Traditional Chinese medicine

## Abstract

**Background:**

Membrane nephropathy (MN) often presents as nephrotic syndrome with characteristic lipid metabolism that could not be explained by lipid indicators commonly used in clinical practice. Studies have shown that invigorating spleen and qi, activating blood and detoxication in the treatment of MN is an effective method proved by randomized controlled clinical trial. However, the alterations of lipid profile before and after traditional Chinese medicine (TCM) treatment and the related lipid markers that affect the therapeutic effect have not been fully clarified.

**Methods:**

We analyzed plasma lipid profiles of 92 patients with MN before and after TCM treatment by high-coverage targeted lipidomics.

**Results:**

675 lipids were identified, of which 368 stably expressed lipids (coefficient of variation less than 30% and deletion value less than 10%) were eventually included for statistical analysis. 105 lipids were altered mainly including spingolipids, glycerides, glycerophosholipid, fatty acyl and steroids, among which, the abundance of ceramides (Cers), sphingomyelins (SMs), diacylglycerols (DGs), phosphatidylcholines (PCs) were lower than those before treatment with statistically significant difference. The WGCNA network to analyze the correlation between the collective effect and the therapeutic effect showed that the triglyceride (TG) molecules were most relevant to the therapeutic effect. Analysis of 162 triglyceride molecules showed that 11 TGs were significantly down-regulated in the effective group which were concentrated in carbon atom number of 52–56 and double bond number of 0–4. TGs molecules including TG56:2-FA20:0, TG56:2-FA20:1, TG56:3-FA20:0 and TG56:5-FA20:2 were most closely related to the therapeutic effect of TCM after adjusting the influence of clinical factors. ROC curve analysis showed that these four lipids could further improve the predictive efficacy of treatment based on clinical indicators.

**Conclusion:**

Our work demonstrated that the therapeutic effect of invigorating spleen and qi, activating blood and detoxication in the treatment of MN may be exerted by regulating lipid metabolism. High-coverage targeted lipidomics provided a non-invasive tool for discovery of lipid markers to improve the predictive efficacy of TCM therapy.

## Introduction

Chronic kidney disease (CKD) is an important global health problem, which often coexists with cardiovascular diseases and lead to poor prognosis of patients [1]. The lipid profile induced by CKD is different from that of the general population [2, 3]. Studies have shown that in addition to the quantitative changes, CKD patients also have a large number of qualitative lipid changes that are not detectable by the lipid indicators used in clinical practice [4], which may result in the high risk of cardiovascular diseases and all-cause mortality. Membranous nephropathy (MN) is a common pathological type of CKD characterized by the deposition of immune complexes containing antigen, IgG, and complement on the subepithelial side of the glomerular basement membrane (GBM) [5]. MN accounts for about 50% of the elderly nephrotic syndrome, which is characterized by obvious lipid disorders complicated with hyperlipidemia.

In recent decades, the field of omics analysis has made significant progress. Lipidomics, as an important constituent of metabonomics, emerged to global study of lipids, including pathways and networks in biological systems [6]. Lipids have diverse biological functions, such as energy storage, structural components of plasma membrane, and primary messengers of cell surface receptors [7, 8]. The wealth of information generated by lipidomics in renal disease enabled us to identify the potential lipid markers associated with renal disease by distinguishing the differences in lipid profiles at different developmental stages. Then, bioinformatics technology was used to analyze the relationship between the changes of its content and the occurrence and development of renal disease, and to infer whether it can be used as an indicator for the diagnosis and intervention of renal disease.

Prognosis of MN varies among individuals, with spontaneous remission occurring in about one-third of patients and progression to end-stage renal disease (ESRD) in another one-third of patients [9]. According to risk stratification for disease progression, patients adopt to different treatment measures. An observation period of at least 6 months and conservative treatment including angiotensin converting enzyme inhibitors (ACEI) and angiotensin receptor blocker (ARB) is recommended. If there are nephrotic range of proteinuria and adverse prognostic factors such as interstitial lesions at the first renal biopsy and renal insufficiency, glucocorticoid combined with immunosuppressive therapy is recommended. Although the majority of patients respond to initial immunosuppressive therapy, 10–40% of patients may be resistant to treatment [10]. On the one hand, some cytotoxic agents can aggravate renal damage, on the other hand, patients often experience a long and painful treatment process of disease progression, dose adjustment, or reuse of alternative drugs [11]. Therefore, it is of great significance to explore more effective and less toxic supportive therapy for MN.

Guiding by the theory of TCM, the research group led by Professor Chen Yiping in the Department of Nephrology of Longhua Hospital noted that the main pathogenesis of MN was "deficiency", "blood stasis", "dampness" and "heat", among which the deficiency of spleen and kidney qi was the basic pathogenesis, the accumulation of dampness and heat was the main content of the pathogenesis, and the interaction of blood stasis and water dampness was an important aspect of the pathogenesis." The method of “invigorating qi, promoting blood, and reducing dampness” was established as the main therapeutic principle of MN, and the clinical effect of the method was widely recognized. A prospective multi-center randomized controlled clinical trial in our center showed that the treatment of MN by the method could protect renal function, and it was superior to standard therapy with prednisone and cyclophosphamide in reducing adverse events [12]. With the increasing incidence of MN in recent years, new evidence shows that exposure to polluted environment, toxic and harmful substances and the "drug toxicity" brought by immunosuppressive therapy may be the important factors for the occurrence of MN. Professor Deng Yueyi proposed that the fifth pathogenesis of MN was "poison". Based on the former formula, the detoxification drugs of *Coptidis Rhizoma* and *rhizoma cimicifugae* were added and the treatment principle was optimized to “invigorating spleen and qi, activating blood and detoxication”. Although the clinical effect of this method is certain, the related factors affecting its therapeutic effect have not been fully clarified. In this study, high-coverage targeted lipidomics was used to study the differences of lipid metabolic profiles before and after the treatment of the method and to search for biomarkers related to therapeutic effect, so as to provide theoretical basis for clinical prediction of the method.

## Materials and method

### Participants cohort

The study enrolled 92 patients diagnosed of primary membranous nephropathy by renal biopsy from Longhua Hospital Affiliated to Shanghai University of traditional Chinese Medicine. At initial follow-up, the MN patient was all in CKD 1–3 stage and 24-h proteinuria (24hpro) was greater than 3500 mg. Each subject received TCM treatment of invigorating spleen and qi, activating blood and detoxication and was followed up for 1 year. The demographic and clinical data, such as age, gender, serum creatinine (Scr), haemoglobin (Hb), albumin (Alb), high density lipoprotein (HDL), low density lipoprotein (LDL), triglyceride (TG), cholesterol (TC), uric acid (UA), 24-h urine protein (24hpro), eGFR (CKD–EPI) were recorded. The TCM prescription consisted of the following 17 herbs: Codonopsis Pilosula, Salvia Miltiorrhiza, Rhizoma Atractylodis, White Atractylodes Rhizome, Poria Cocos, Semen Coicis, Pueraria Lobata, Ligusticum Wallichii, Radix Astragali, Rhizoma Dioscoreae, Angelica Sinensis, Radix Glycyrrhizae, Viola Mandshurica, Scutellaria Barbata, Herba Hedyotidis Diffusae, Coptidis Rhizoma and Rhizoma Cimicifugae.

Patients were excluded if they had one of the following conditions: (1) rapid-progressive MN (eGFR decreased by more than 50% within 3 months); (2) secondary membranous nephropathy such as systemic lupus erythematosus and hepatitis B virus associated nephritis; (3) severe diseases or dysfunction of other organs; (4) complicated with serious infection and life-threatening complications; (5) pregnant or lactating women; (6) administration of glucocorticoids, immunosuppressants and other therapeutic drugs during the follow-up period. This study protocol has been approved by the Ethics Committee of Longhua Hospital Affiliated to Shanghai University of Traditional Chinese Medicine (Clinical Study Project No. 2018-008) and has clinicaltrials.gov ID: NCT02610595. Registered on November 20, 2015, https://clinicaltrials.gov/NCT0261059. Each study subject signed an informed consent.

### Therapeutic effect measure

In the study, the effective treatment of TCM outcomes was the composite of complete or partial remission at 1 year. Complete remission was defined as proteinuria less than 300 mg per 24 h and eGFR decreased by less than 15% from baseline. Partial remission was defined as a reduction in proteinuria of at least 50% from baseline and proteinuria less than 3500 mg per 24 h with eGFR decreased by less than 15% from baseline. Failure to meet complete and partial remission criteria was defined as ineffective treatment of TCM.

### Blood collection

The blood samples were collected using EDTA anticoagulant tubes and stored in 4℃ refrigerator until centrifugation (3000 rpm, 15 min, 4℃). The supernatants were partitioned by microcentrifuge tubes and stored in a refrigerator at − 80℃ until lipid extraction was performed.

### Lipid extraction

The modified MTBE (methyl tert-butyl ether) method was used for lipids extraction. The detailed experimental protocol were as following: (1) Taken 10 μL of sample and thawed at room temperature; (2) added 60 μL methanol and vortex to mix; (3) added 200 μL of methyl tertbutyl ether (MTBE) for vigorously vibration; (4) add deionized water 50 μL vortexed for 1 min and centrifuge for 10 min, 2500 g at room temperature; (5) The supernatant (75 μL) was transferred to a new 1.8 mL glass vial and mixed 15 μL multiple internal standards (Sciex, Foster City, CA); (6) evaporated to dryness under gentle nitrogen stream; (6) added 100 μL dichloromethane/methanol (volume ratio: 1:1) solution to reconstitute and taken 25 μL of the supernatant for mass spectrometry analysis.

### LC–MS/MS analysis

Lipidomics analysis was performed by Nexera X2 LC-30AD system (Shimadzu Scientific Instruments, Marlborough, MA) and Sciex 5500 QTRAP Mass Spectrometer (Applied Biosystems/Sciex). We used ACQUITY UPLC BEH HILIC Column (130 Å, 2.1 × 100 mm, 1.7 µm; Waters Corp) for chromatographic separation. Analyst 1.6.3 software (AB Sciex, Foster City, CA) was applied for data acquisition. Plasma samples were randomly analyzed on LC–MS and quality control samples were interspersed between every 10 samples to assess repeatability and stability. Sample injection volume in both ionization modes was 5 μL. The sample and column temperature were respectively maintained at 4 °C and 40 °C. The mobile phases containing 50:50 (v/v) acetonitrile/water with 10 mM ammonium acetate (pH 8.0) (A) and pure acetonitrile (B) were used for eluting lipids at flow rate of 300 μL/min. The gradient elution program was performed as follow: 0.1 min, 85% B; 7.5 min, 65% B; 8.5 min, 5% B; 11 min, 5% B; 11.1 min, 85% B; 15 min, 85% B.

Mass spectrometry analysis was performed in positive and negative mode using electrospray ionization. Multiple reaction monitoring (MRM) mode was used for data acquisition. The curtain gas was set as 35 psi and collision gas was set as medium. The pressure of ion source gas 1 was 40 psi and the pressure of gas 2 was 50 psi. The ion spray voltage is 5.5 kV in positive mode and − 4.5 kV in negative mode. Source gas temperature was set at 500 °C.

### Statistical analysis

Quantification was performed by calculating the peak area ratio of the identified transitions to their corresponding stable isotope-labeled internal standard (IS) using MultiQuant 3.0.2 software (Sciex, Foster City, CA).

Descriptive statistics (median (P25, P75)) was used to describe characteristics of the MN patients. Wilcoxon’s Signed Rank test was used for continuous variables and chi-squared test was applied for categorical variables to compare baseline characteristics between effective and ineffective group.

Paired Wilcoxon’s Signed Rank test was used to compare the clinical indicators and lipid data before and after treatment. Missing values of lipid data were imputed as half of the minimum value, because the missing values were lower than the minimum limit of detection. Missing values of clinical data were imputed as mean values. The cross-sectional associations of lipid molecules (scaled to SD of 1) with baseline 24hpro and albumin were analyzed by multivariate linear regression and visually displayed by forest plot.

Weighted gene co-expression network analysis (WGCNA) was employed to classify modules of highly interconnected lipids which were defined by topological overlap measure (TOM). The network plot was performed by Cytoscape [13]. Logistic regression model was used to analyze the relative risk (RR) and 95% confidence interval (CI) for therapeutic effect of TCM according to quartile and per SD increment of lipid module and each lipid after adjustment for different clinical indicators. Lipids were considered significant when corrected with Benjamini & Hochberg (BH) correction in the adjusted model. Receiver operating characteristic (ROC) curve was used to calculate the area under the curve combined with different indexes.

Statistical analyses were used by SPSS 21.0 and R386 4.0.3 software. All *P* values were 2 sided and *P* value less than 0.05 was considered statistically significant.

## Results

### Characteristics of participants

#### Comparison of clinical indicators before and after treatment

A total of 92 MN patients were enrolled in this study and their clinical indicators before and after treatment were shown in Table 1. After one year of treatment with the method of invigorating spleen and qi, activating blood and detoxication, we found that 24hpro was significantly lower and serum albumin was significantly higher in MN patients than those at baseline (*P* < 0.0001). Compared with before treatment, lipid levels such as LDL and CHOL improved and the difference was statistically significant (*P* < 0.0001). After12-month treatment, the renal function of the MN patients was stable at normal level compared with the baseline, and there was no significant difference between the two groups (*P* > 0.05) (Table 1).

**Table 1:** Comparison of clinical indicators of 92 MN patients before and after treatment

Indicators	Before treatment	After treatment	*Z*	*P*
Hb (g/L)	119.5 (108.0, 136.0)	121 (112.5, 134.3)	− 0.191	0.848
Alb (g/L)	26.8 (20.6, 30.0)	28.8 (24.5, 35.5)	− 6.404	< 0.0001
HDL (mmol/L)	1.2 (1.0, 1.6)	1.1 (0.9, 1.3)	− 3.558	< 0.0001
LDL (mmol/L)	4.2 (3.2, 5.8)	3.3 (2.7, 4.2)	− 4.723	< 0.0001
TG (mmol/L)	2.4 (1.6, 3.4)	1.9 (1.4, 2.6)	− 1.931	0.053
CHOL (mmol/L)	6.5 (5.3, 8.1)	5.2 (4.4, 6.1)	− 4.925	< 0.0001
Scr (μmol/L)	61.9 (51.9, 75.7)	65.4 (49.5, 77.1)	− 0.526	0.599
UA (μmol/L)	372 (292.3, 416.5)	379 (306, 423)	− 1.620	0.105
24hpro (g/24 h)	4.189 (3.5, 5.1)	3.2 (1.0, 4.3)	− 5.998	< 0.0001
eGFR (mL/min/1.73m^2^)	99.7 (87.6, 110.0)	98.2 (81.6, 108.0)	− 0.205	0.838

#### Comparison of baseline indicators in different therapeutic effect groups

92 MN patients were grouped according to the therapeutic effect of TCM after one year of treatment. Eighteen patients had a complete remission and 26 patients had a partial remission. In the total 92MN patients, the treatment was effective in 44 patients, and the total effective rate was 47.8%. There was no significant difference in baseline clinical indicators between the two different therapeutic effect groups (*P* > 0.05) (Table 2).

**Table 2:** Comparison of baseline indicators in different therapeutic effect groups

Indicators	Effective group (N = 44)	Ineffective group (N = 48)	*Z/χ*^2^	*P*
Sex (male%)	24 (53.3%)	27 (56.3%)	0.080	0.778
Age (y)	56 (38, 66)	60 (51.5, 67.5)	− 1.427	0.154
Hb (g/L)	126 (109, 142)	121 (110, 136)	− 0.628	0.530
Alb (g/L)	27.3 (23.4, 30)	25 (18.7, 30.0)	− 1.772	0.076
HDL (mmol/L)	1.2 (1.1, 1.8)	1.21 (1.0, 1.6)	− 0.594	0.552
LDL (mmol/L)	4.2 (3.1, 4.9)	4.54 (3.2, 6.1)	− 1.061	0.289
TG (mmol/L)	2.1 (1.5, 3.2)	2.5 (1.8, 3.5)	− 1.581	0.114
CHOL (mmol/L)	6.3 (5.1, 7.0)	7.3 (5.5, 8.9)	− 1.757	0.079
Scr (μmol/L)	58.4 (48.1, 75.7)	65.6 (56.4, 76.65)	− 1.403	0.161
UA (μmol/L)	377 (284, 452)	365 (293.5, 412.5)	− 0.511	0.609
24hpro (g/24 h)	4.1 (3.4, 5.2)	4.2 (3.3, 5.1)	− 0.008	0.994
eGFR (mL/min/1.73m^2^)	103.5 (89.9, 114.0)	98.4 (87.1, 106.7)	− 1.714	0.086

### Analysis of lipidomics

#### Results of lipid identification

**Fig. 1:**
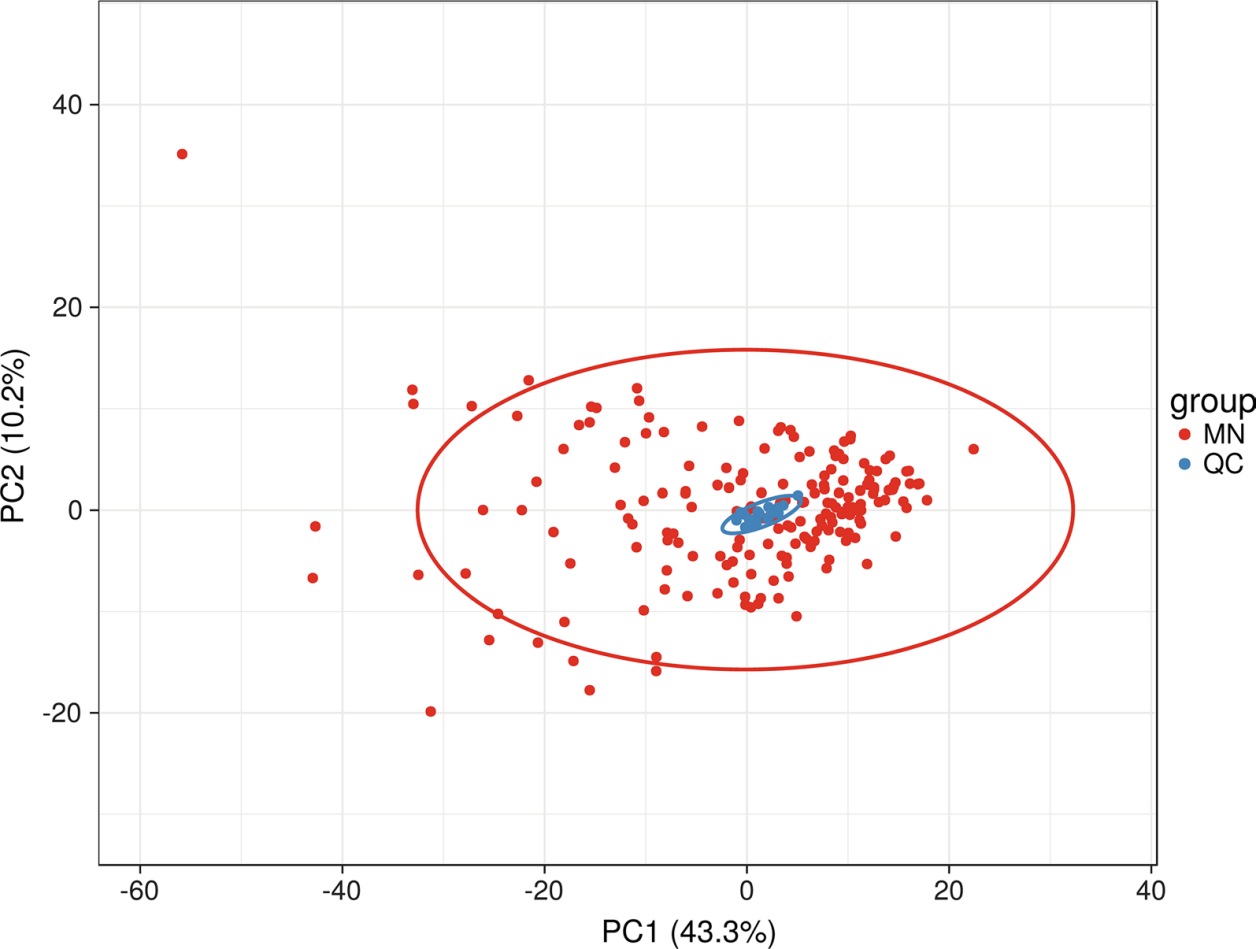
Principal component analysis of test sample and QC sample

184 lipid samples were collected from 92 MN patients before and after treatment. There were 19 quality control (QC) samples and every 10 samples separated by one QC sample. A total of 675 lipids were identified by high-coverage targeted lipidomics, of which 368 lipids with coefficient of variation (CV) less than 30% and deletion value less than 10% were included for the final statistical analysis. The 368 lipids identified were classed into 10 main groups. Triglyceride (TG), accounted for the highest proportion (44.02%), followed by phosphatidylethanolamine (PE) (17.93%), phosphatidylcholine (PC) (13.86%), sphingomyelin (SM) (11.14%), fatty acid (FA) (3.80%), lysophosphatidylcholine (LPC) (3.53%), diacylglycerol (DG) (1.90%), cholesterol ester (CE) (1.90%), ceramide (Cer) (1.36%), phosphatidylserine (PS) (0.54%). Principal component analysis (PCA) on all test samples and QC samples was performed. The results showed that the quality control of samples were clustered together and located in the middle of each group, indicating good repeatability of the experiment (Fig. 1).

#### Cross-sectional association between lipids and 24hpro, albumin at baseline

**Fig. 2:**
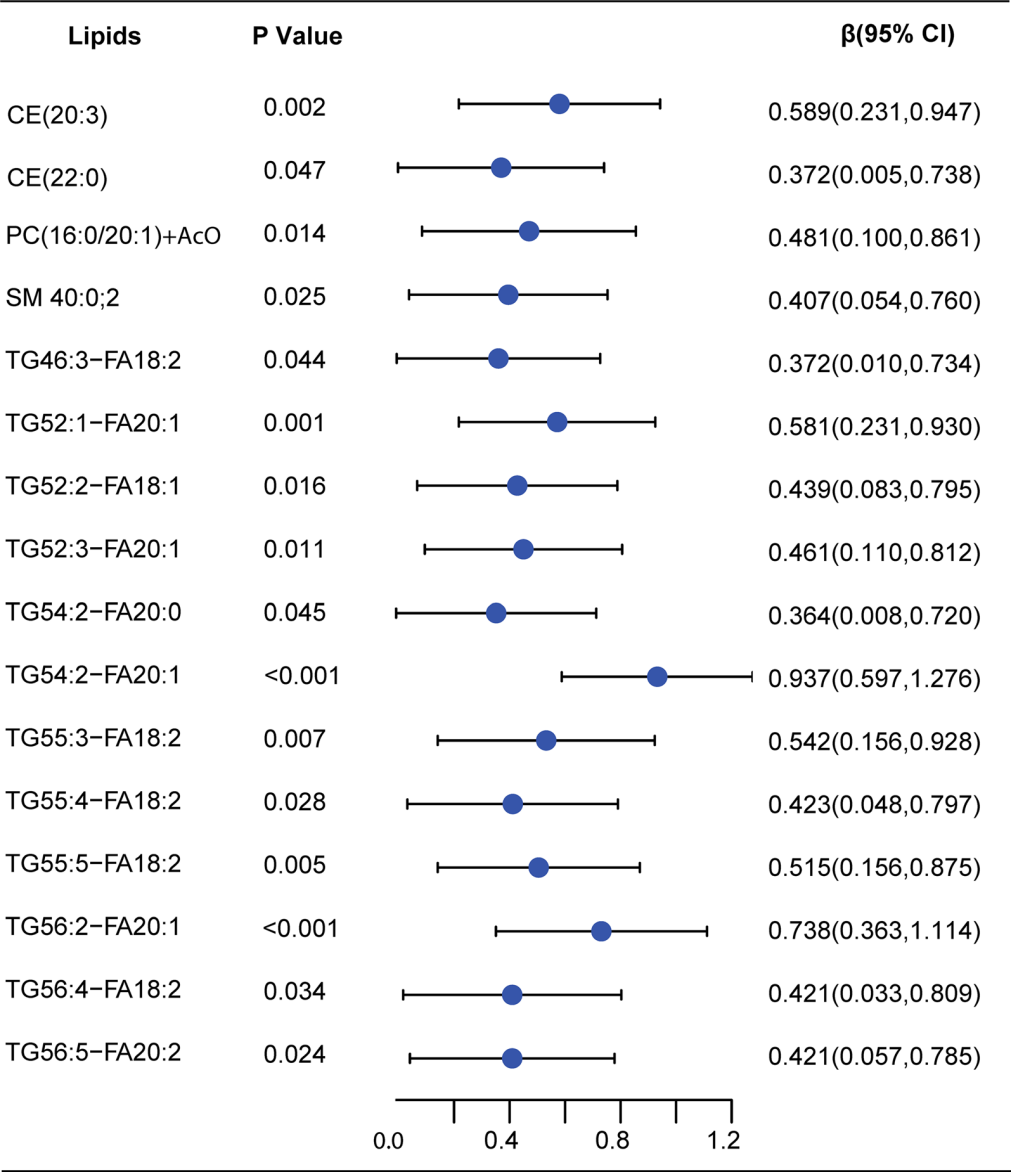
Forest plot for baseline lipid moleculars and 24hpro correlation analysis

**Fig. 3:**
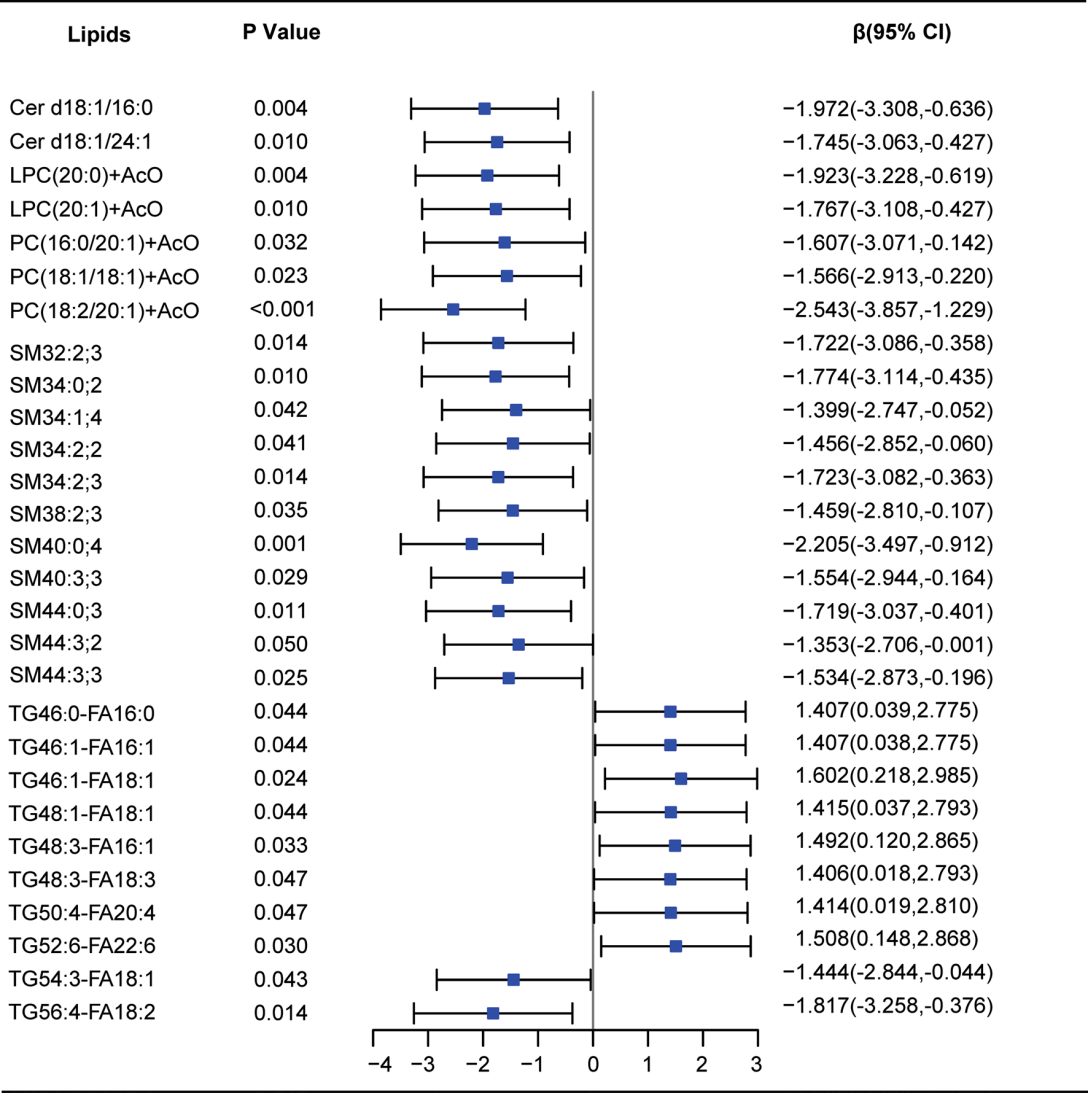
Forest plot for baseline lipid moleculars and albumin correlation analysis

In cross-sectional analysis at baseline, 16 lipids species including CE (20:3), CE (22:0), PC (16:0/20:1), SM 40:0; 2, TG46:3-FA18:2, TG52:1-FA20:1, TG52:2-FA18:1, TG52:3-FA20:1, TG54:2-FA20:0, TG54:2-FA20:1, TG55:3-FA18:2, TG55:4-FA18:2, TG55:5-FA18:2, TG56:2-FA20:1, TG56:4-FA18:2, TG56:5-FA20:2were positively and nominally significantly associated with baseline 24hpro after adjusting for sex, age, hypertension, diabetes, creatinine, uric acid, albumin. There were 28 lipids moleculars including 5 classes (Cers, LPCS, PCs, SMs, TG-FAs) that were associated with albumin after adjustment for sex, age, hypertension, diabetes, creatinine, uric acid, and 24hpro. Among them, Cers, LPCS, PCs, SMs, and TG54:3-FA18:1, TG56:4-FA18:2 were negatively and nominally significantly correlated with albumin, whileTG46:0-FA16:1, TG46:1-FA16:1, TG48:1-FA18:1, TG48:3-FA18:1, TG48:3-FA18:3, TG50:4-FA20:4, TG52:6-FA22:6, were positively and nominally significantly correlated with albumin (Figs. 2, 3).

#### Alterations of lipids after treatment with TCM

**Fig. 4:**
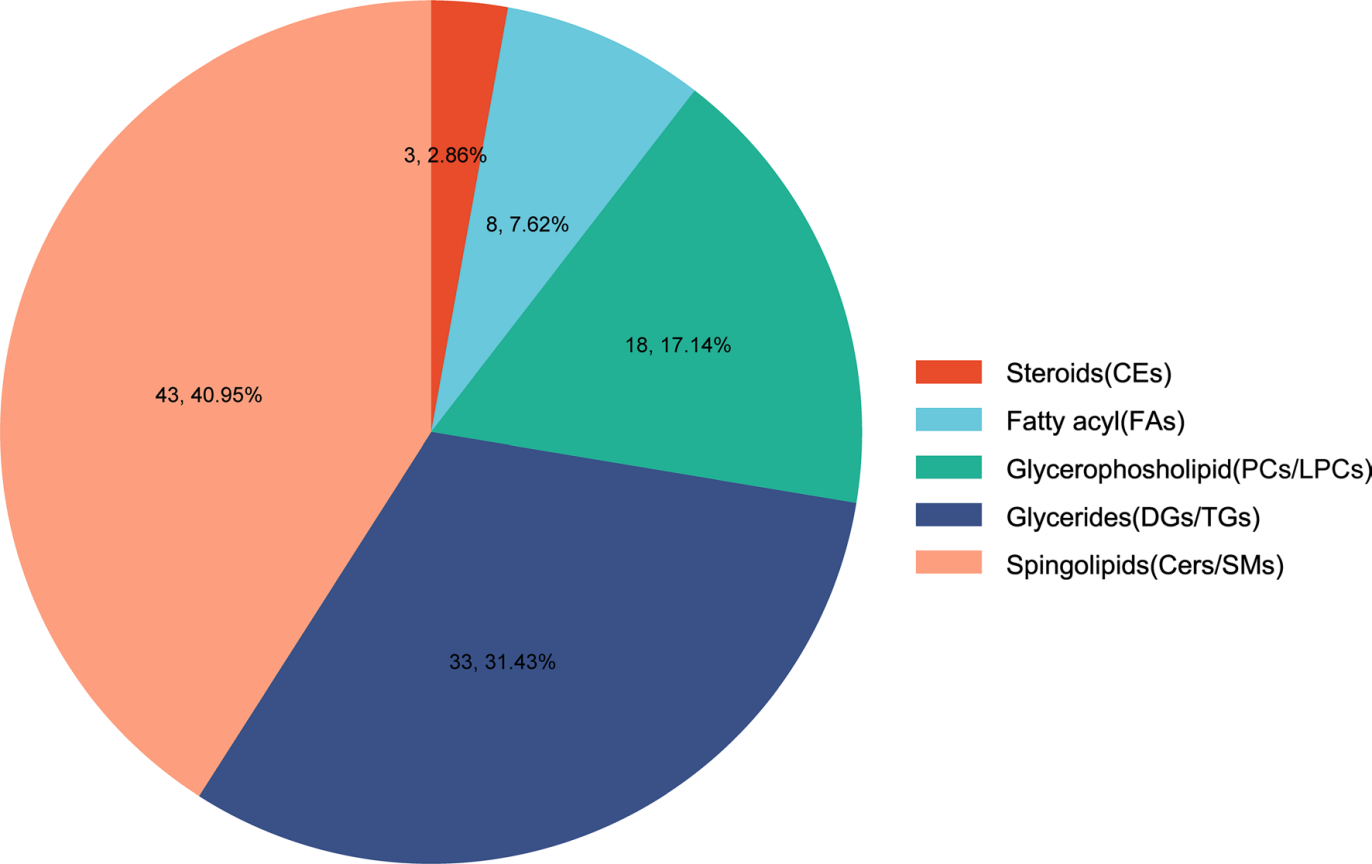
The lipid categories with statistical difference before and after TCM treatment

**Fig. 5:**
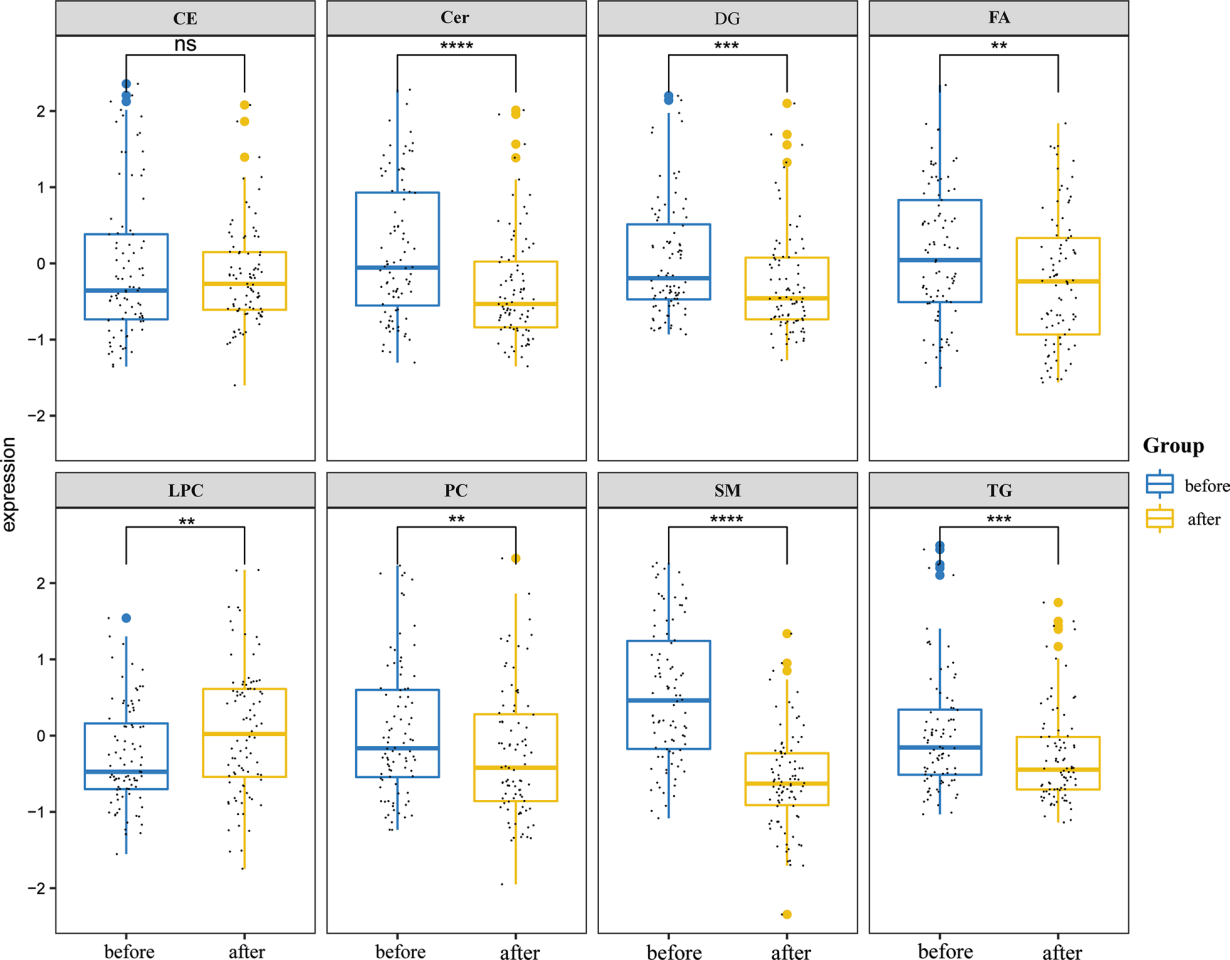
Boxplot of alteration in lipid abundance before and after TCM treatment

A total of 105 lipid molecules achieved Benjamini & Hochberg (BH)-level significance were screened after one year of treatment with the prescription of invigorating spleen and qi, activating blood and detoxication. They were mainly divided into 43 spingolipids (Cers/SMs), 33 glycerides (DGs/TGs), 18 glycerophosholipid (PCs/LPCs), 8 fatty acyl and 3 steroids (Fig. 4). Among them, sphingolipids, glycerides and fatty acyl were significantly down-regulated, while LPCs were significantly up-regulated and PCs were significantly down-regulated in glycerophosholipid (Fig. 5).

#### Differential lipid molecules were analyzed by WGCNA network

**Fig. 6:**
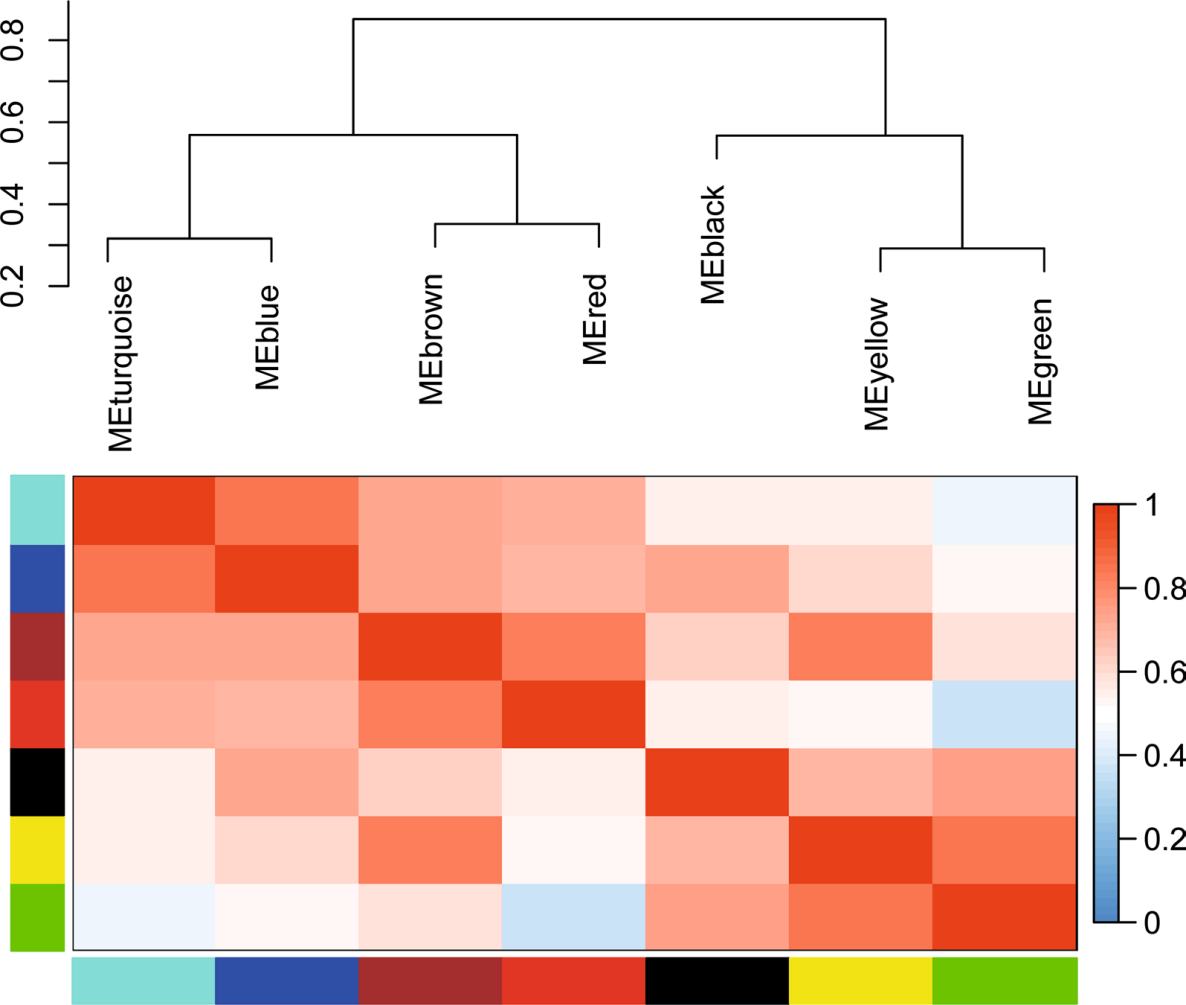
Correlation heatmap among the 7 modules. (Different color bars on the left and at the bottom represent different modules, and gradient color on the right indicate Pearson correlation coefficients among modules)

**Fig. 7:**
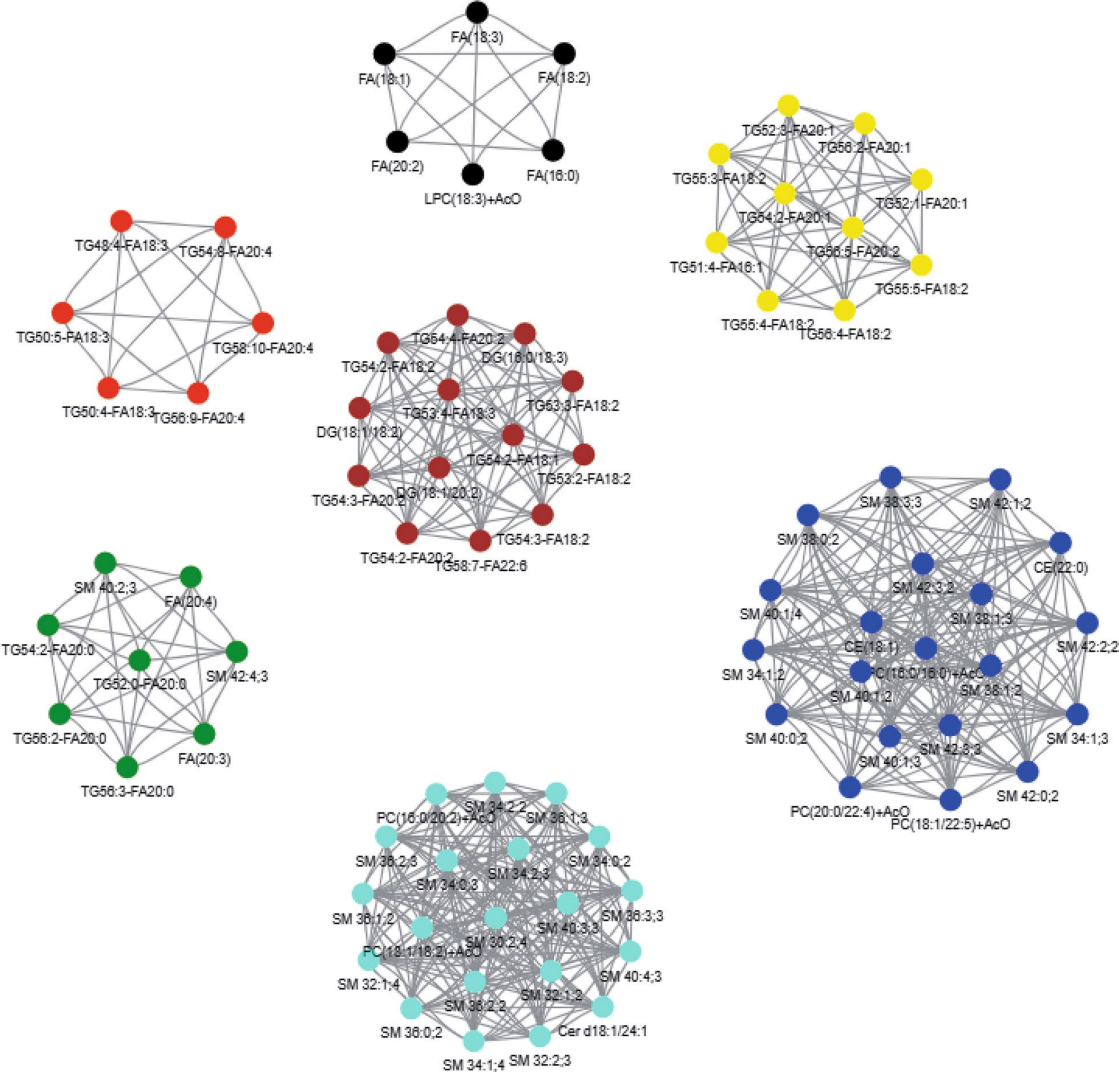
7 different color network plots represent the modules detected by topological overlap measure

To determine the collective effect among highly correlated lipid modules, WGCNA analysis was performed on 105 lipid molecules that differed before and after treatment and 7 modules were detected by topological overlap measurement. The red module consisted of six TGs with carbon atom number of 48–58 and double bond number of 4–10. The green module comprised of four TGs with carbon atomic number 52–56 and double bond number 0–2, two FAs and two SMs molecules. The yellow module was mainly composed of TGs with carbon atom number of 51–56 and double bond number of 2–5. Brown module included DGs and TGs molecules with carbon atomic number 53–58 and double bond number 2–7. Turquoise module mainly composed of sphingolipids (SMs/Cer) and PCs molecules. The blue module comprised of SMs, PCs and CEs molecules (Figs. 6, 7).

To further explore the correlation between different modules and the therapeutic effect of TCM. Model 1 adjusted for gender, age, diabetes and hypertension and Model 2 adjusted for albumin, creatinine, uric acid and 24hPro on the basis of Model 1. The results showed that the yellow module was correlated with the therapeutic effect of TCM in the models. However, when the relative concentration of yellow module was divided by quartile, the correlation trend was reduced and the correlation trend was no longer statistically significant (*P* > 0.05). The green module showed a high correlation with the treatment effect of TCM (*P* value < 0.05). Compared with the low quartile, the high quartile significantly reduced the probability of effective treatment of TCM (*P* trend < 0.05) (Table 3). It was analyzed that the yellow and green modules were mainly TG lipid molecules, so the correlation between TG lipid molecules and therapeutic effect would be further explored in the following sections.

**Table 3:** Correlation between different modules and therapeutic effect of TCM

Module	Quartiles of module eigengenes				*P* trend	RR per SD	*P* value
	Q1	Q2	Q3	Q4			
Module yellow; number of molecules = 10							
case/total	15/22	10/22	10/23	8/23			
Model 1	1	0.341 (0.096, 1.217)	0.279 (0.076, 1.025)	0.215 (0.058, 0.787)	0.106	0.003 (0.000, 0.282)	0.042
Model 2	1	0.290 (0.077, 1.083)	0.234 (0.060, 0.911)	0.212 (0.056, 0.810)	0.089	0.003 (0.000, 0.282)	0.045
Module green; number of molecules = 8							
Case/total	15/22	12/22	10/23	6/23			
Model 1	1	0.473 (0.130, 1.715)	0.374 (0.107, 1.307)	0.124 (0.031, 0.492)	0.028	0.002 (0.000, 0.133)	0.029
Model 2	1	0.537 (0.140, 2.056)	0.344 (0.096, 1.234)	0.118 (0.028, 0.505)	0.031	0.001 (0.000, 0.110)	0.026
Module turquoise; number of molecules = 40							
Case/total	8/22	12/22	13/23	10/23			
Model 1	1	1.954 (0.549, 6.956)	2.513 (0.720, 8.771)	1.225 (0.354, 4.236)	0.449	1.500 (0.026, 86.403)	0.898
Model 2	1	1.689 (0.452, 6.319)	3.080 (0.832, 11.398)	1.406 (0.391, 5.050)	0.386	2.721 (0.042, 176.870)	0.852
Module red; number of molecules = 6							
Case/total	9/22	11/22	13/23	10/23			
Model 1	1	1.376 (0.407, 4.653)	1.614 (0.475, 5.482)	0.853 (0.244, 2.991)	0.718	2.390 (0.040, 141.941)	0.898
Model 2	1	1.319 (0.351, 4.952)	1.476 (0.410, 5.307)	0.455 (0.111, 1.862)	0.323	0.453 (0.005, 40.559)	0.852
Module brown; number of molecules = 13							
Case/total	10/22	12/22	11/23	10/23			
Model 1	1	1.599 (0.455, 5.616)	1.021 (0.299, 3.489)	0.777 (0.223, 2.712)	0.725	0.135 (0.002, 9.505)	0.830
Model 2	1	1.615 (0.434, 6.005)	0.736 (0.204, 2.659)	0.672 (0.181, 2.491)	0.582	0.069 (0.001, 6.060)	0.565
Module blue; number of molecules = 20							
Case/total	9/22	12/22	12/23	10/23			
Model 1	1	2.103 (0.600, 7.370)	1.648 (0.472, 5.754)	1.086 (0.319, 3.692)	0.611	4.102 (0.072, 232.322)	0.863
Model 2	1	2.097 (0.579, 7.592)	1.520 (0.421, 5.496)	1.091 (0.310, 3.838)	0.658	3.931 (0.060, 256.329)	0.852
Module black; number of molecules = 6							
Case/total	11/22	9/22	11/23	12/23			
Model 1	1	0.779 (0.224, 2.706)	0.934 (0.269, 3.242)	0.995 (0.295, 3.357)	0.977	1.302 (0.023, 74.558)	0.898
Model 2	1	0.837 (0.230, 3.036)	1.011 (0.274, 3.732)	0.958 (0.273, 3.361)	0.992	0.804 (0.012, 52.620)	0.918

Model 1 adjusted for gender, age, diabetes and hypertension. Model 2 adjusted for albumin, creatinine, uric acid and 24hPro on the basis of Model 1. RR, relative risk

#### TG lipid molecules and structures in different therapeutic effect groups

**Fig. 8:**
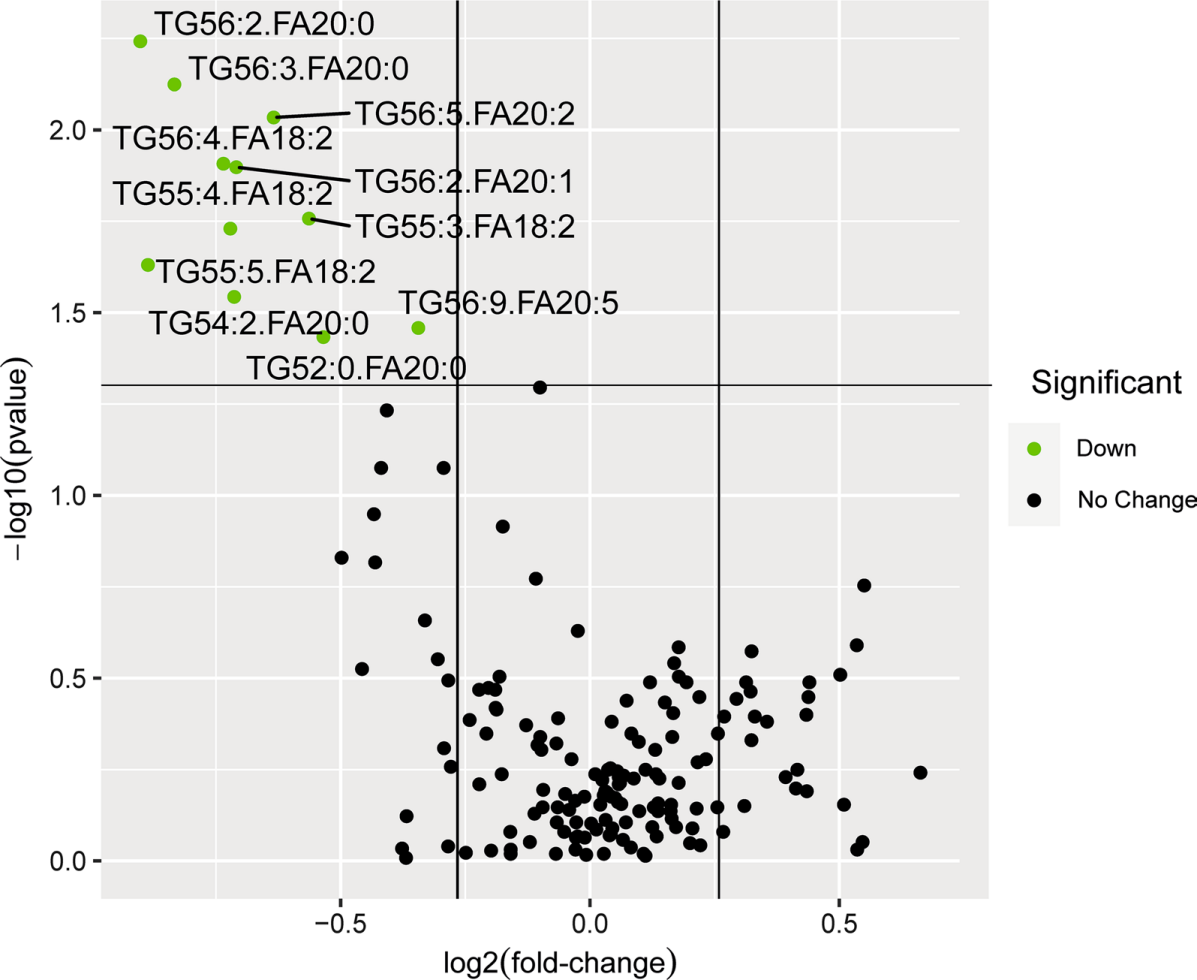
Volcano plot analysis of baseline lipid species with significant changes in different therapeutic effect groups (The significance level was *P* < 0.05 and the ploidy change value (|log2 (fold change) | > 0.26). Down: the relative abundance of the lipids was significantly lower than those in ineffective group. No change: the relative abundance of the lipids had no significant difference.)

**Fig. 9:**
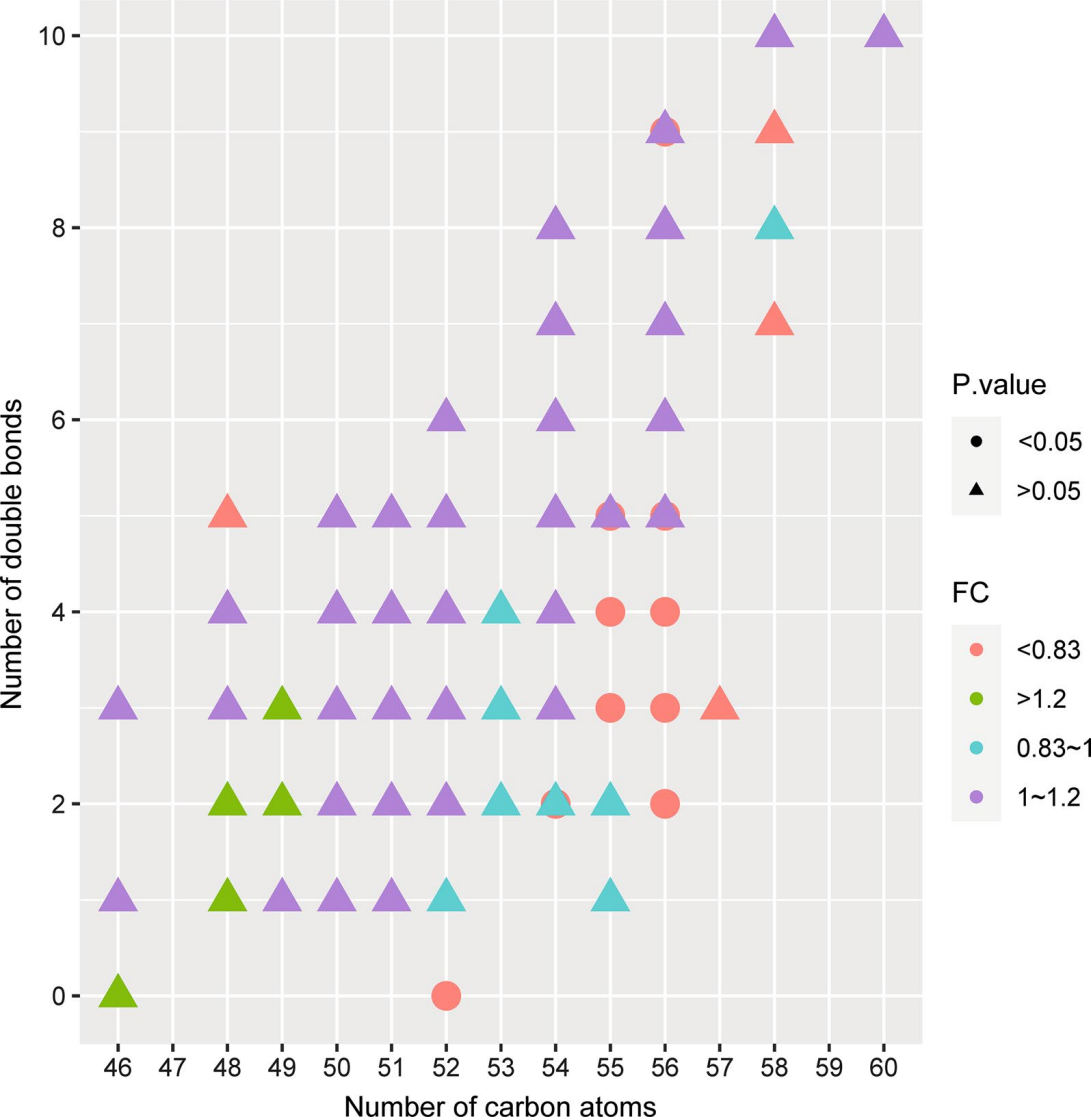
Ballplot analysis of structures of TGs lipid molecules in different therapeutic effect groups (Dots represent significance less than 0.05, and different colors represent different fold change expression)

A total of 162 TGs at baseline were analyzed based on whether the treatment of TCM was effective. 11 lipids were found to be significantly down-regulated in the effective group (*P* < 0.05, Fold Change < 0.83). The 11 lipids included TG52:0-FA20:0, TG54:2-FA20:0, TG55:3-FA18:2, TG55:5-FA18:2, TG55:5-FA18:2, TG56:2-FA20:0, TG56:2-FA20:1, TG56:3-FA20:0, TG56:4-FA18:2, TG56:5-FA20:2, TG56:9-FA20:5 (Fig. 8). The structures of fatty acid chain of TG lipid molecules with significantly different therapeutic effects were further explored. The results showed that the 11 significantly down-regulated TG molecules concentrated in carbon atom number of 52–56 and double bond number of 0–4 (Fig. 9).

#### Correlation between 11 lipid molecules and clinical indicators

**Fig. 10:**
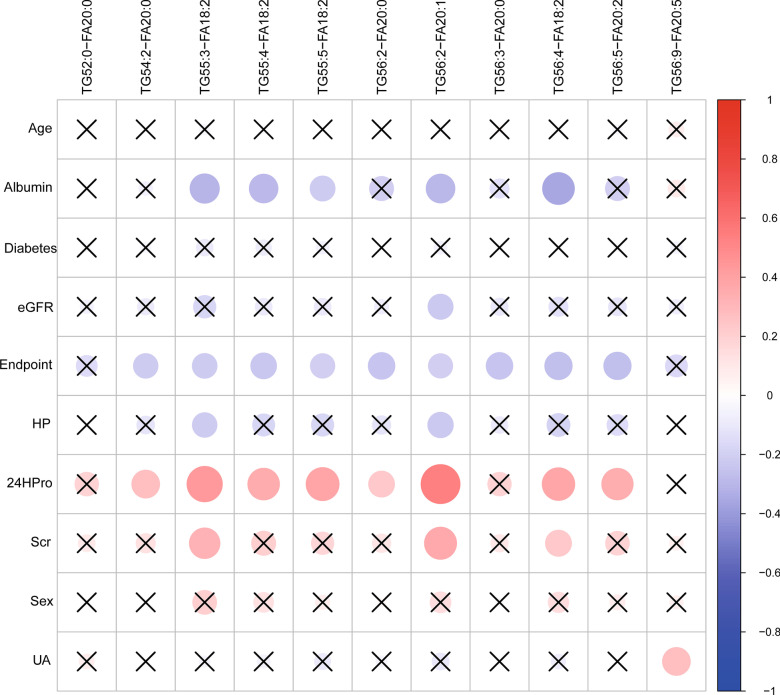
The correlation between 11 lipids selected and clinical indicators (○ represents the correlation is statistically significant. The size of ○ represents the correlation coefficient, × represents the correlation is not statistically significant)

The correlation between lipids can better understand the degree of association between different lipids, and roughly predict whether the molecular biological activity of different lipids is correlated. The results showed that the correlations among the 11 lipid molecules were positively statistically significant. The correlation between lipid molecules and clinical indicators showed that the 8 lipid molecules, TG56:2-FA20:0, TG56:5-FA20:2, TG:56:4-FA18:2, TG56:2-FA20:1,, TG55:4-FA18:2, TG55:3-FA18:2, TG55:5-FA18:2, TG54:2-FA20:0, were positively correlated with 24HPro. Five lipid molecules, TG56:4-FA18:2, TG56:2-FA20:1, TG55:4-FA18:2, TG55:3-FA18:2, TG55:5-FA18:2, were negatively correlated with albumin. The pairwise correlations of 11 lipid molecules and their correlations with clinical indicators were shown in Fig. 10.

#### Correlation analysis of 11 lipid molecules with therapeutic effect

**Fig. 11:**
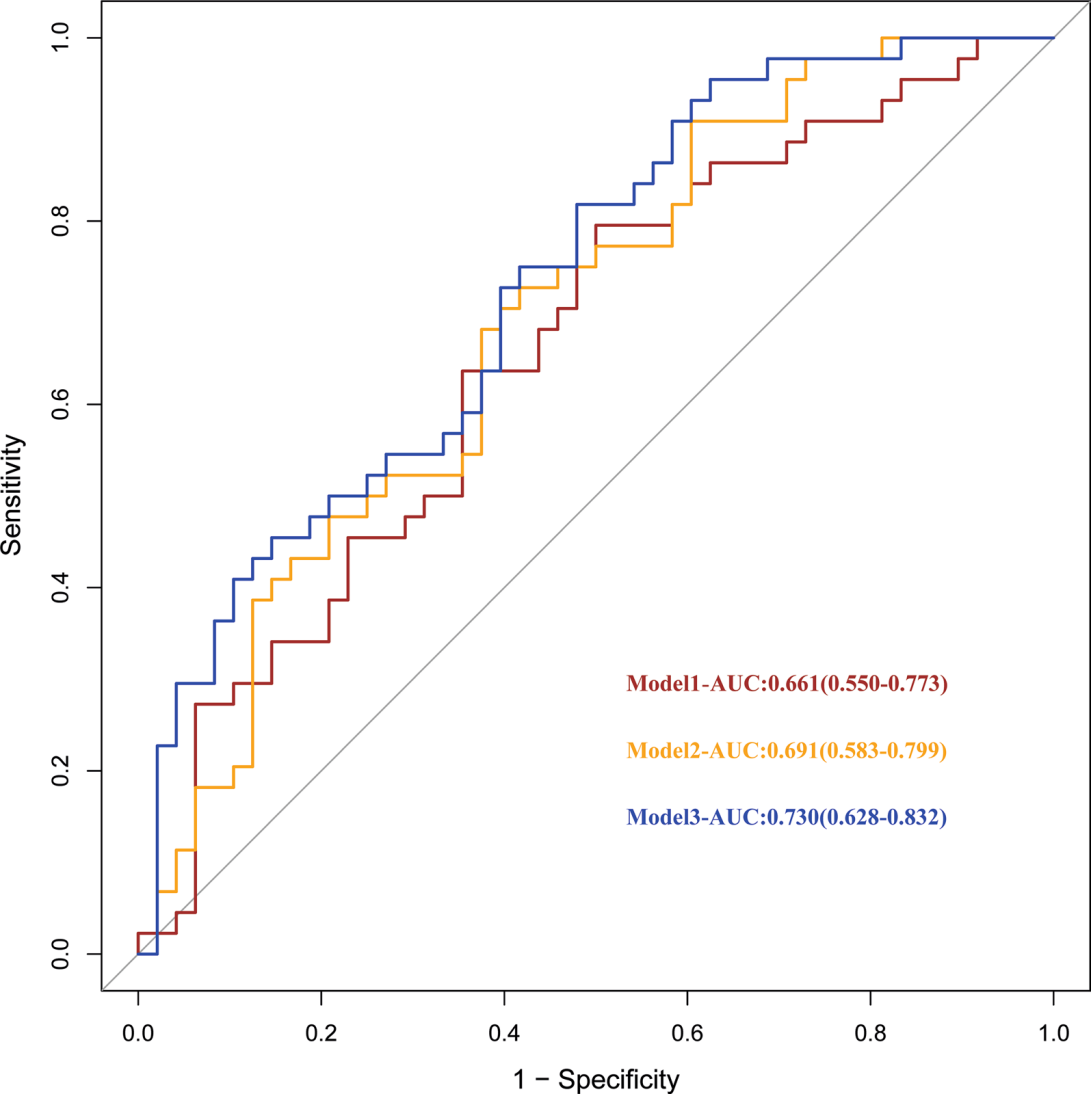
ROC curve for prediction of therapeutic effect of TCM. (Model 1: Adjusted for age, gender, hypertensive, diabetes, uric acid, creatinine, albumin, 24hpro; Model 2: Adjusted for Model 1, HDL, LDL, TG, CHOL; Model 3: Adjusted for Model2, TG56:2-FA20:0, TG56:2-FA20:1, TG56:3-FA20:0, TG56:5-FA20:2)

Four different logistic regression models were constructed to explore the correlation between lipids and therapeutic effect by adjusting different clinical indicators. Model 1 was adjusted for gender, age, hypertension and diabetes; Model 2 was adjusted for albumin, creatinine, uric acid and 24 h proteinuria on the basis of Model 1; Model 3 was adjusted for hemoglobin and urea nitrogen on the basis of Model 2. Model 4 further adjusted HDL, LDL, TG and CHOL on the basis of Model 3. The results showed that the top 10 lipids were related to the therapeutic effect in Model 1, 2, 3. When TG, CHOL, HDL and LDL were added into the adjustment model, only four lipid molecules, TG56:2-FA20:0, TG56:2-FA20:1, TG56:3-FA20:0 and TG56:5-FA20:2, were correlated with the therapeutic effect with statistical significance (Table 4). Receiver operating characteristic (ROC) curve was analyzed to further investigate the predictive efficacy of selected lipids on therapeutic effect. Model 1 adjusted for gender, age, hypertension, diabetes, uric acid, creatinine, and albumin, the area under the curve was 0.661 (95% CI 0.550–0.773). When HDL, LDL, TG and CHOL were added to Model 1, the area under the curve increased to 0.691 (95% CI 0.583–0.799). Model 3 added four lipid molecules including TG56:2-FA20:0, TG56:2-FA20:1, TG56:3-FA20:0 and TG56:5-FA20:2 related to therapeutic effect on the basis of Model 2, the area under the curve increased to 0.730 (95% CI 0.682–0.832), which further improved the prediction efficiency of therapeutic effect of TCM (Fig. 11).

**Table 4:** Correlation between 11 lipids selected and therapeutic effect of TCM

Name	Model 1		Model 2		Model 3		Model 4	
	OR (95% CI)	*P*	OR (95% CI)	*P*	OR (95% CI)	*P*	OR (95% CI)	*P*
TG52:0-FA20:0	0.602 (0.385, 0.941)	**0.026**	0.575 (0.360, 0.920)	**0.021**	0.577 (0.359, 0.926)	**0.023**	0.636 (0.384, 1.053)	0.078
TG54:2-FA20:0	0.594 (0.382, 0.922)	**0.020**	0.573 (0.362, 0.906)	**0.017**	0.572 (0.360, 0.910)	**0.018**	0.629 (0.384, 1.028)	0.064
TG55:3-FA18:2	0.565 (0.353, 0.904)	**0.017**	0.573 (0.352, 0.933)	**0.025**	0.581 (0.350, 0.963)	**0.035**	0.624 (0.354, 1.099)	0.103
TG55:4-FA18:2	0.557 (0.349, 0.888)	**0.014**	0.565 (0.349, 0.914)	**0.020**	0.568 (0.346, 0.931)	**0.025**	0.619 (0.362, 1.061)	0.081
TG55:5-FA18:2	0.581 (0.369, 0.916)	**0.019**	0.575 (0.358, 0.922)	**0.022**	0.578 (0.357, 0.935)	**0.026**	0.624 (0.376, 1.034)	0.067
TG56:2-FA20:0	0.537 (0.339, 0.851)	**0.008**	0.533 (0.332, 0.856)	**0.009**	0.532 (0.330, 0.860)	**0.010**	0.576 (0.347, 0.958)	**0.033**
TG56:2-FA20:1	0.517 (0.322, 0.832)	**0.007**	0.529 (0.327, 0.855)	**0.009**	0.528 (0.322, 0.865)	**0.011**	0.554 (0.316, 0.971)	**0.039**
TG56:3-FA20:0	0.540 (0.337, 0.865)	**0.010**	0.541 (0.332, 0.881)	**0.014**	0.536 (0.328, 0.877)	**0.013**	0.586 (0.350, 0.981)	**0.042**
TG56:4-FA18:2	0.522 (0.324, 0.842)	**0.008**	0.536 (0.328, 0.876)	**0.013**	0.529 (0.319, 0.878)	**0.014**	0.573 (0.325, 1.010)	0.054
TG56:5-FA20:2	0.565 (0.353, 0.904)	**0.007**	0.520 (0.318, 0.849)	**0.009**	0.515 (0.312, 0.850)	**0.009**	0.540 (0.306, 0.953)	**0.033**
TG56:9-FA20:5	0.557 (0.349, 0.888)	0.131	0.612 (0.368, 1.016)	0.057	0.617 (0.367, 1.036)	0.068	0.688 (0.356, 1.330)	0.266

Bold means *p* value ≤ 0.05

Model 1: Adjusted for sex, age, hypertension, and diabetes

Model 2: Adjusted for Model 1, albumin, creatinine, uric acid, 24hpro

Model 3: Adjusted for Model 2, Hb and urea nitrogen

Model 4: Adjusted for Model 3, HDL, LDL, TG, CHOL

## Discussion

The MN samples before and after treatment with the method of invigorating spleen and qi, activating blood and detoxication were analyzed by high-coverage targeted lipidomics, and the following conclusions were drawn: (1) After TCM treatment, 105 lipids were altered from those before treatment, mainly including spingolipids, glycerides, glycerophosholipid, fatty acyl and steroids. Among them, the abundance levels of Cers, SMs, DGs, PCs were lower than those before treatment, and the difference was statistically significant. (2) The WGCNA network was used to analyze the correlation between the collective effect and the therapeutic effect of lipids with differences before and after treatment. The results showed that the TGs molecule was most relevant to the therapeutic effect. (3) Based on the therapeutic effect, 162 TGs molecules at baseline were analyzed. The results showed that 11 lipid molecules were significantly down-regulated in the effective group, and the 11 significantly down-regulated TGs molecules were concentrated in carbon atom number of 52–56 and double bond number of 0–4.4) TGs molecules including TG56:2-FA20:0, TG56:2-FA20:1, TG56:3-FA20:0 and TG56:5-FA20:2 are most closely related to the therapeutic effect of TCM after adjusting the influence of clinical factors. ROC curve analysis showed that these four lipids could further improve the predictive efficacy of treatment based on clinical indicators.

Patients with MN are often clinically presented as nephrotic syndrome, which induces lipid disorders manifested as an accumulation of total cholesterol, markedly increased LDL cholesterol, Non-HDL cholesterol and triglycerides. With the development of lipidomics, the use of conventional lipid measurements to describe the lipoprotein abnormality in CKD is insufficient for early CKD diagnosis, monitoring and the prediction of treatment. Lipidomics can detect dynamic alterations in lipid metabolism that occur before changes in renal function or kidney tissues detectable changes. Studies have shown that the accumulation of total fatty acids, glycerides, and glycerophosholipid in patients with CKD is directly associated with increased serum triglyceride levels and is inversely associated with eGFR [14]. In addition, the detection of some of the lipid molecules, such as diacylglycerol (32:0, 36:0) and monoacylgycerol 16:0, can improve the efficiency of eGFR and urinary protein creatinine ratio (UPCR) in predicting CKD stage 3 progression to ESRD [15]. A cross-sectional study of dyslipidemia associated with ischemic stroke in CKD revealed a significant difference between phosphatidylcholine (PC) and phosphatidylethanolamine (PE) lipids in the stroke and non-stroke patients with CKD. After adjusting for age, blood pressure, history of cardiovascular disease, the use of statin, and eGFR, the model showed that PC 38:4 and FFA16:0 were associated with an increased risk of ischemic stroke with CKD [16]. A study of the association between glycosylated sphingolipids and progression to CKD in type 1 diabetes showed that the reduction of long chain lactosylceramides was significantly associated with an increased risk of progression to macroalbuminuria, but not with kidney dysfunction. In hexosylceramides, the only significant association observed was an increase in C18:1-H associated with an increased risk of CKD progression. In summary, a large body of evidence supported that the role of lipid molecules and lipid-derived metabolites in the pathogenesis of renal disease. Therefore, the analysis of key lipid mediators has become an important tool in the diagnosis, prognosis and treatment of renal disease.

Chinese herbal consisted of complex compounds has the characteristics of multiple components, multiple targets and overall regulation. Systems biology is featured as systematicness, wholeness and dynamism, which is based on genome, proteome and metabolome, providing new ideas and approaches for the study of TCM. Some studies have shown that lipidomics, as a branch of metabolomics, can well reflect the alteration in lipid metabolism profile after treatment with TCM and further verified that TCM played a role in treatment of disease by regulating lipid metabolism pathway. Fang-ying Xia et al. analyzed 27 acylcarnitines before and after TCM treatment of IgA nephropathy and found that some short and median chain acylcarnitines were independently associated with baseline eGFR and eGFR changes after TCM treatment. Patients with high C5:1, C8:1, C3DC, C10:1 and C5DC at baseline had worse prognosis and response to TCM treatment. This study suggested that acylcarnitine could be used as a biomarker for prognosis and treatment response of IgA nephropathy treated with TCM to some extent [17]. Another study investigated the correlation between different types and structures of lipid molecules and prognosis of IgA nephropathy treated with TCM. Results showed that medium chain triglycerides containing less unsaturated fatty acids (≤ 3 double bonds) were positively related to better prognosis of IgA nephropathy patients with TCM treatment and long chain polyunsaturated fatty acids (PUFAs) in DG, LPC, LPE, PC, PE, PI, and TG were positively relevant to better renal prognosis in patients with the combined treatment of corticosteroids and TCM. This study provides a new idea and theoretical basis for prognosis judgment and precision treatment of IgA nephropathy treated by TCM.

In the study, we found certain Cers and SMs of sphingolipids were significantly down-regulated after treatment with TCM. Ceramides (Cers) are usually formed by combining a sphingosine group with a fatty acid residue and can be re-synthesized in the endoplasmic reticulum from dietary fatty acids and serine. Subsequently, sphingolipids (SM) can be formed by adding a polar head to ceramides. SMs accounts for about 87% of plasma sphingolipids and is a major lipoprotein phospholipid, which can transduce transmembrane signals and is widely involved in apoptosis, proliferation, differentiation, inflammation, oxidative stress, etc. It was reported that SMs was associated with a higher risk of atherosclerosis and coronary heart disease [18]. The effect of endogenous sphingolipids on renal disease is manifested as low concentration of glycosylated end products promoting the synthesis of sphingolipid product S1P in the glomerulus, leading to the proliferation of glomerular mesangial cells in the diabetic nephropathy model [19]. Zhong et al. showed that TCM can exert the therapeutic effect on IgA nephropathy by regulating mesangial cell proliferation induced by sphingolipid metabolism [20]. Our study suggested that TCM may play a therapeutic role in MN by downregulating lipid disorders of Cers and SMs in sphingolipids.

Studies have shown that abnormalities in glycerophosholipid metabolism can be observed in patients with advanced CKD. PC and LPC belong to glycerolipids. LPCs acted on G-protein-coupled receptors, mainly showing pro-atherosclerotic activity. But LPCs have also been reported to have antibacterial, antioxidant and anti-atherosclerotic effects [21]. Low levels of LPCs were thought to be associated with acute kidney injury, CKD, sepsis, and poor prognosis [22, 23]. Other studies have shown no change or increase in LPCS levels in patients with CKD [14]. The level of serum PC in patients with CKD compared to normal controls is also controversial. Some studies reported a significant decrease of PC levels in HD patients, while another study showed a tendency to increased serum PC levels in patients with CKD [24, 25]. The different results may be caused by the different structures of the regulated PC or LPC lipid molecules. This study showed that the level of LPCs was significantly up-regulated and the level of PCs was significantly down-regulated compared with those before treatment.

Studies have shown that compared with early CKD, patients with advanced CKD are characterized by increased saturated free fatty acid (FFA) abundance, which can result in a series of adverse consequences, including autophagy, apoptosis, cell death, and eventually lead to the progression of CKD through mediating mechanisms of endoplasmic reticulum stress. In addition, the utilization of FFAs and the mechanisms for regionalization of complex lipids were activated, including up-regulation of sterol-CoA desaturase-1 (SCD1) and DAG acyltransferase1 (DGAT1), leading to the synthesis of TAG. With the further progression of CKD, β-oxidation disorders are prevalent, which will lead to the accumulation of free fatty acids in the cell, thus forming a vicious cycle of damage [26]. Another study has shown that lipid-induced the downregulation of mitochondrial fatty acid β-oxidation and inhibition of AMP kinase (AMPK) activity lead to endoplasmic reticulum stress, autophagy, and apoptosis in podocytes, endothelial cells, and proximal tubular epithelial cells. Mitochondrial dysfunction causes cell death in glomerular endothelial cells and podocytes, leading to proteinuria, glomerular inflammation, and glomerulosclerosis. Apoptosis of proximal tubular epithelial cells results in interstitial inflammation and ultimately interstitial fibrosis. Inflammation further inhibits AMPK activity, causing a vicious feedforward cycle of lipid toxicity that amplifies renal injury [27]. Network analysis of our study showed that FA (20:3) and FA (20:4) were included in the green modules related to the therapeutic effect, indicating that the increase of the abundance of these two FFA affected the exertion of the therapeutic effect of TCM. The method of invigorating spleen and qi, activating blood and detoxication can significantly down-regulate the serum FFAs in patients with MN, which may regulate the lipid disorders of the body to some extent by regulating the FFA β oxidation, autophagy and apoptosis in podocytes, endothelial cells, and proximal tubular epithelial cells, AMPK activity, and endoplasmic reticulum stress.

Triglycerides belong to glycerides, which are the most abundant lipids in the human body. The types of triglyceride molecules are directly related to atherosclerosis, diabetes, obesity, stroke and other diseases. In a population-based study, Stegemann et al. identified a group of specific triglyceride clusters with saturated and monounsaturated acyl chains that were strongly associated with cardiovascular disease [28]. A recent Chinese study showed that some triglyceride molecules with 48–60 carbon atoms and double bond number less than 5 are inversely associated with the risk of developing type 2 diabetes [29]. The exploration of triglyceride lipid molecules in renal disease focused on the relationship between molecular structure and disease progression. The study by Eugene P. Rhee et al. showed that compared with the control group of normal renal function population, the pattern of triacylglycerol containing 40–48 carbons was significantly lower and triacylglycerol containing higher carbon numbers was increased in ESRD patients, and this heterogeneity was masked by standard measurements of total triglycerides [30]. Another study investigating changes in triglyceride abundance with different CKD stages showed that from CKD2 to CKD5, the abundance of high-carbon polyunsaturated triglycerides increased, while that of low-carbon triglycerides with low double bond numbers decreased [26]. Most existing studies covered a small number of triglyceride lipid molecules, our study included 162 triglyceride molecules. The results showed that TCM treatment could reduce the level of triglyceride, which were most related to the therapeutic effect in the WGCNA analysis of the collective effect related to the therapeutic effect. 11 significantly down-regulated TG molecules in effective group concentrated in carbon atom number of 52–56 and double bond number of 0–4. After adjusting the clinical indicators, it was found that TG56:2-FA20:0, TG56:2-FA20:1, TG56:3-FA20:0 and TG56:5-FA20:2 were most correlated with the therapeutic effect and could increase the predictive efficiency of the therapeutic effect. The results showed that the different molecules in each kind of lipids showed different biological characteristics due to the different number of carbon atoms, double bonds and the saturation position.

In general, there are relatively few studies using lipidomics to explore the mechanism of action of TCM and they mainly focus on animal experiments. This study provides an analytical idea for the clinical study of high-coverage targeted lipidomics to explore the target of TCM treatment of MN which revealed the profound changes of lipid metabolites. Next, we will conduct animal experiments to further clarify the ways by which the method of invigorating spleen and qi, activating blood and detoxication can regulate lipid metabolism and play a therapeutic role, so as to provide a new upstream target for the treatment of membranous nephropathy.

The results showed that invigorating spleen and qi, activating blood and detoxication may played a therapeutic role by regulating lipid metabolism, in which TG lipid molecules can improve the predictive efficacy of TCM treatment. However, there are still some limitations in our study. First of all, no normal control group was established in the design of this study. Comparing the difference of lipid metabolism profiles between patients with MN and normal people is helpful to further determine whether the effect of TCM is exerted through the regulation of abnormal lipids. Secondly, in addition to sampling and experimental factors, the dynamic nature of biological processes may be another important reason for the difficulty in reproducibility of biomarker discovery. Therefore, the four potential lipid markers found in this study that could improve the efficacy prediction of TCM treatment still need to be further verified in large cohort and clinical trials. Finally, although our analysis largely reflected the dynamic characteristics of lipids in the body, it only explained the association between changes in metabolites and disease, rather than causation.

## Conclusion

Our study demonstrated the correlation between lipid molecules and clinical indicators and therapeutic effect of TCM by regulating lipid metabolism. The role of collective effect and individual effect of lipids in predicting the therapeutic effect of TCM were explored. Four lipid molecules were identified which could further improve the efficacy of predicting the therapeutic effect on the basis of clinical measurement. High-coverage targeted lipidomics provided a non-invasive tool for predicting the efficacy of TCM treatment of MN. With the development of systems biology, the focus of the research has shifted to the acquisition of omics data, emphasizing the interaction between biological networks and organs. The multivariate integration of lipidomics with other omics and dynamic monitoring at different time points can maximize the utility of omics data.

## Data Availability

The data and materials used in the current study are available from the corresponding author on reasonable request.
